# DTDP-rhamnosyl transferase RfbF, is a newfound receptor-related regulatory protein for phage phiYe-F10 specific for *Yersinia enterocolitica* serotype O:3

**DOI:** 10.1038/srep22905

**Published:** 2016-03-11

**Authors:** Junrong Liang, Xu Li, Tao Zha, Yuhuang Chen, Huijing Hao, Chang Liu, Ran Duan, Yuchun Xiao, Mingming Su, Xin Wang, Huaiqi Jing

**Affiliations:** 1 National Institute for Communicable Disease Control and Prevention, Chinese Center for Disease Control and Prevention, State Key Laboratory of Infectious Disease Prevention and Control, Collaborative Innovation Center for Diagnosis and Treatment of Infectious Diseases, No.155, Changbai Road, Changping, Beijing, 102206, China; 2 Wuhu Municipal Centre for Disease Control and Prevention, No. 178, Jiuhua central Road, Wuhu, Anhui Province, 241000, China; 3 Department of Pathogenic Biology, School of Medical Science, Jiangsu University, Xuefu Road, Zhenjiang, Jiangsu Province, 212013, China; 4 Institute of Biophysics, Chinese Academy of Sciences, No. 15, Datun Road, Chaoyang, Beijing, 100101, China

## Abstract

Bacteriophages and their hosts are continuously engaged in evolutionary competition. Here we isolated a lytic phage phiYe-F10 specific for *Yersinia enterocolitica* serotype O:3. We firstly described the phage receptor was regulated by DTDP-rhamnosyl transferase RfbF, encoded within the *rfb* cluster that was responsible for the biosynthesis of the O antigens. The deletion of DTDP-rhamnosyl transferase RfbF of wild type O:3 strain caused failure in phiYe-F10 adsorption; however, the mutation strain retained agglutination with O:3 antiserum; and complementation of its mutant converted its sensitivity to phiYe-F10. Therefore, DTDP-rhamnosyl transferase RfbF was responsible for the phage infection but did not affect recognition of *Y. enterocolitica* O:3 antiserum. Further, the deletions in the putative O-antigen biosynthesis protein precursor and outer membrane protein had no effect on sensitivity to phiYe-F10 infection. However, adsorption of phages onto mutant HNF10-Δ*O-antigen* took longer time than onto the WT, suggesting that deletion of the putative O-antigen biosynthesis protein precursor reduced the infection efficiency.

The primary determinant in the infection of a bacterial host by a bacteriophage is the adsorption of the phage to the host receptor. This receptor recognition is the most reported in phage- host interaction studies^1^,^2^. *Yersinia enterocolitica* is Gram-negative and a globally distributed foodborne human pathogen, belonging to the Enterobacteriaceae *Yersinia* species. There are about 60 different serotypes with variability in the O-antigen. *Y. enterocolitica* clinical isolates from humans predominantly belong to serotypes O:3, O:9, O:8 and O:5, 27 with variability on different continents^3^,^4^: serotypes O:3 and O:9 cause human infections and are most common in Europe, Canada, Japan, China and South Africa; while 1B/O:8 is the primary serotype infecting people in the Americas^5^,^6^. However, at present, serotype O:3 strains are becoming the most frequently detected pathogenic *Y. enterocolitica* all over the world^7^,^8^,^9^,^10^.

To date, the phage phiYeO3-12 and vB_YenP_AP5 display specificity for *Y. enterocolitica* O:3 and phage PY54 exhibits a host range restrict to *Y. enterocolitica* O:5 and O:5, 27 were previously described^11^,^12^,^13^,^14^,^15^. Phage viruses have stringent host specificities where the attachment of the virus particle requires specific recognition of phage receptor using a phage receptor binding protein (RBP)^16^,^17^. Several receptors are reported, including flagella/pilus related components^18^,^19^, lipopolysaccharides (LPS)^20^,^21^,^22^, capsular polysaccharides (CPS) and outer membrane proteins (OMP)^23^,^24^. Similar to other Gram-negative Enterobacteriaceae bacteria, the structure of the *Yersinia enterocolitica* LPS has three primary components: lipid A, core oligosaccharide and O-side-chain (O-antigen). LPS acts as an immune stimulatory agent (lipid A) or as a virulence factor (O-antigen)^25^,^26^,^27^. The lipid A portion is responsible for the endotoxin activity. The O-antigen functions as a barrier against complement-mediated lysis and resists killing of bacteria by microbicidal intracellular granules in polymorpho-nuclear leucocytes. Previously published assays showed that we can detect the serotypes of *Y. enterocolitica* strains using amplification of O-antigen-encoded genes^28^. The *Yersinia enterocolitica* O:3 lipopolysaccharide O-antigen is a homopolymer of 6-deoxy-L-altrose. The genes for the O:3 O-antigen translocation are located within the *rfb* gene cluster, including 10 open reading frames and eight of the genes, are organized into two operons, *rfb*ABC and *rfb*DEFGH that are essential for O-antigen synthesis^26^. A specific detection of *Y. enterocolitica* serotype O:3 is obtained with fragment of the *rfbC* gene^29^. In this study, we evaluated the lysis ability of the phage phiYe-F10 on the *Y. enterocolitica* wild strain, a spontaneous rough mutant, three gene deletion strains and a *rfbc* compensation strain using phage adsorption tests and plaque formation tests. We are first to identify DTDP-rhamnosyl transferase RfbF as the receptor regulator protein, and another protein (putative O-antigen biosynthesis protein precursor) in a *Y. enterocolitica* O:3 strain is related to phiYe-F10 adsorption.

## Results

### The host range of phage phiYe-F10

The 188 *Y. enterocolitica* strains were (Supplemental data 1): 57 strains serotype O:3, 34 strains serotype O:9, 13 strains serotype O:8, 10 strains serotype O:5, 3 strains serotype O:5, 27, 5 strains of self-agglutinating *Y. enterocolitica* and 66 other serotypes biotypes 1A *Y. enterocolitica* (Among the six *Y. enterocolitica* biotypes, 1A is the most heterogeneous group, including more than 17 different serotypes, some of them are unable to serotyped^30^). At 25 °C and 37 °C, phiYe-F10 can formed plaques only on serotype O:3 strains, but not on *Y. enterocolitica* strains other O serogroups, or *Y. pseudotuberculosis* or *Y. pestis* strain (Table 1). All of the 57 sensitive strains were O:3 serotype *Yersinia enterocolitica* carrying the DTDP-rhamnosyl transferase RfbF encoding genes (*rfbc*), 49 of which were pathogenic *Y. enterocolitica* (including 39 strains biotype 3, 6 strains biotype 4, and 4 strains of biotype 5); 6 strains were biotype 1A nonpathogenic; and 2 strains were biotype 1A with *ail* genes (Table 2).

### Morphology

**Figure 1:**
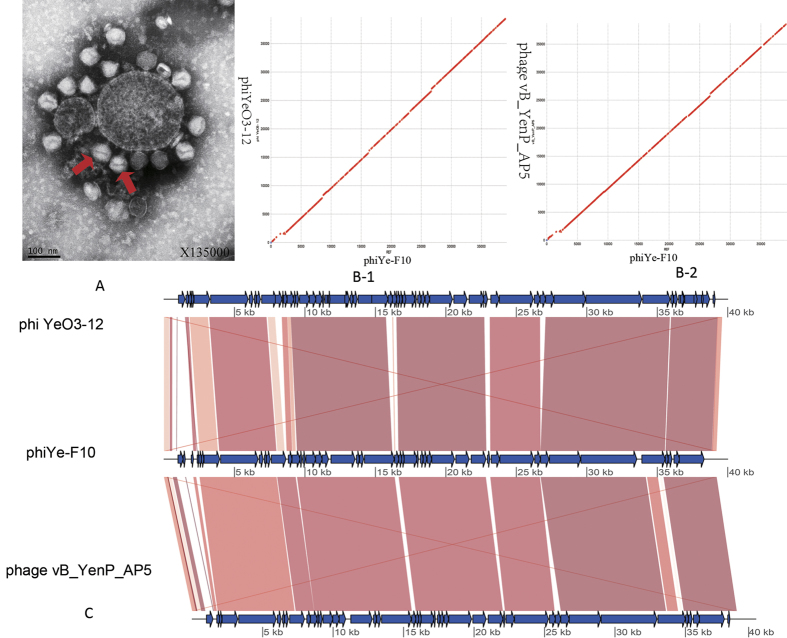
(**A**) Electron micrograph of phiYe-F10. The phage is negatively stained with 2% potassium phosphotungstate. phiYe-F10 is shown at 135,000× magnification. Scale bar indicates size in nm. (**B-1**) Dot plot of genome sequences of phiYe-F10 and *Yersinia* phage phiYeO3-12. (**B-2**) Dot plot of genome sequences of phiYe-F10 and *Yersinia* phage vB_YenP_AP5. (**C**) Pairwise nucleotide sequence comparison of phiYe-F10, phiYeO3-12 and vB_YenP_AP5.

phiYe-F10 was negatively stained and examined using transmission electron microscopy (Fig. 1A showed with an arrow). The virions showed hexagonal outlines, indicating their icosahedral nature. The head connected with the tail through a short neck which exhibiting T7 symmetry in shape. The phage had a head at approximately 55.0 nm in diameter and a short non-contractile tail of ~11.0 nm in length. Extended tail fibers were not seen. Collectively, these morphological features indicated that this virus belongs to the family *Podoviridae*.

### General features of the phiYe-F10 genome and comparative genomics

The phiYe-F10 genome was assembled as a circular molecule when the sequencing was completed. Using PCR, the results of terminal run-off sequencing confirmed that the phiYe-F10 has repeated sequences. The genomes of T7-like phages typically contain direct terminal repeats (DTRs) that are used during genome replication and packaging. For example, *Yersinia* phage phiYeO3-12 (Genbank No. NC_001271.1) and vB_YenP_AP5 (Genbank No. NC_025451.1) have DTRs of 232 bp and 235 bp, respectively. The lengths of the DTRs in phiYe-F10 (235 bp) were in agreement with the reported lengths of T7-like phage members. So we concluded the DNA sequence of the phiYe-F10 consists of a linear double stranded DNA of 39,210 bp, which correlates well with the size of other T7-like phage members. In total, 46 gene products were predicted in the phiYe-F10 genome; functions were assigned to 43 of them based on the similarities of the predicted products to known proteins.

**Figure 2:**
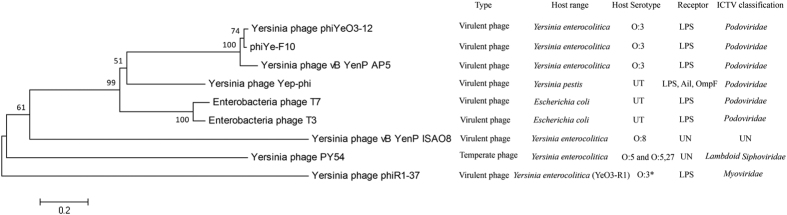
The phylogenetic tree of tail fiber proteins. *Strain YeO3-R1, a virulence-plasmid-cured O antigen-negative derivative of *Yersinia enterocolitica* serotype O:3. The phage receptor of phi R1-37 is in the outer core hexasaccharide of *Y. enterocolitica* O:3 LPS. UT: untyped; UN: unknown.

Compared with the other two lytic phages for *Y. enterocolitica* serotype O:3, the genome of phiYe-F10 was highly similar to the 39,600 bp of *Yersinia* phage phiYeO3-12 (97%, 623/641) and the 38,646 bp of *Yersinia* phage vB_YenP_AP5 (89%, 583/648). Genomic comparisons indicated phages phiYe-F10, phi YeO3-12 and vB_YenP_AP5 were closely related. A dot plot comparison using the program Gepard was performed to illustrate the genomic similarities among phiYe-F10, phi YeO3-12 and vB_YenP_AP5. The red dots indicated the corresponding genome regions on the abscissa and the ordinate showed similarity (Fig. 1B-1,B-2). We also performed a graphical comparison of the three genome sequences. Highly related sequences were shown using red shadings. As shown in Fig. 1C, the genome sequence of phiYe-F10 had exactly similar genetic organization and large blocks of homologous synteny when compared to the other two phages. The differences were primarily for the 403 bp and 446 bp deletions (with the region from 26,675 bp to 27,078 bp of *Yersinia* phage phiYeO3-12 and the 25,710 bp to 26,156 bp of vB_YenP_AP5 deleted in the genome sequence of phiYe-F10). The missing genes encoded putative 13.5 protein for phage phiYeO3-12; encoded hypothetical protein and portion of internal virion protein A for phage vB_YenP_AP5. The Fig. 1C data was in concordance with the Fig. 1B-1,1 B-2 data, strongly indicated that phiYe-F10, phage phiYeO3-12 and vB_YenP_AP5 were closely associated.

For bacteriophages, the primary determinant of the host receptor is the tail fiber protein, so we compared of the tail fiber protein of *Yersinia* phage which including the families *Podoviridae*, *Myoviridae* and *Siphoviridae*. The alignment showed high homology among phiYe-F10, phiYeO3-12 and vB_YenP_AP5. The six families *Podoviridae* members were classified into the same cluster, the other sequences of the families *Myoviridae* and *Siphoviridae* divided from that of the families *Podoviridae*, and located in different branches (Fig. 2).

### The adsorption abilities and growth characteristics of the WT strain, its mutants, and compensation strain

Early studies showed the outer membrane protein A (OmpA) was not only a common phage receptor of Enterobacteriaceae^23^,^31^, but also a common immune protective antigen beyond the V antigen conserved within *Yersinia* species^32^,^33^. The phage Yep-phi, required not only LPS but also Ail and OmpF for its efficient infection, losing of the *ail* and *ompF* products may resulted in defective phage receptors^34^. In order to identified whether OmpA was involved in the phage infection of phiYe-F10, we constructed of HNF10-Δ*ompA* knock-out mutants to test the growth characteristic and phage adsorption efficiency. The O-side-chain of *Yersinia enterocolitica* O:3 is a homopolymer of 6-deoxy-L-altrose^35^. Our research had suggested that the *Yersinia enterocolitica* serogroup O:3 were all carried with the fragment of the *rfbC* gene. The *rfbc* gene clustered in the *rfb* locus of the bacterial chromosome, and encoded the dTDP -rhamnosyl transferase RfbF. It directed the O-antigen biosynthesis through synthesize the O-antigen glycosyltransferases, which transferred the rhamnosyl to growing repeat units. As the phage phiYe-F10 specific for *Y. enterocolitica* serotype O:3, and the *rfbc* sequence was strictly found in sensitive strains, we wanted to identify whether dTDP -rhamnosyl transferase RfbF was involved in the phage infection of phiYe-F10.

**Figure 3:**
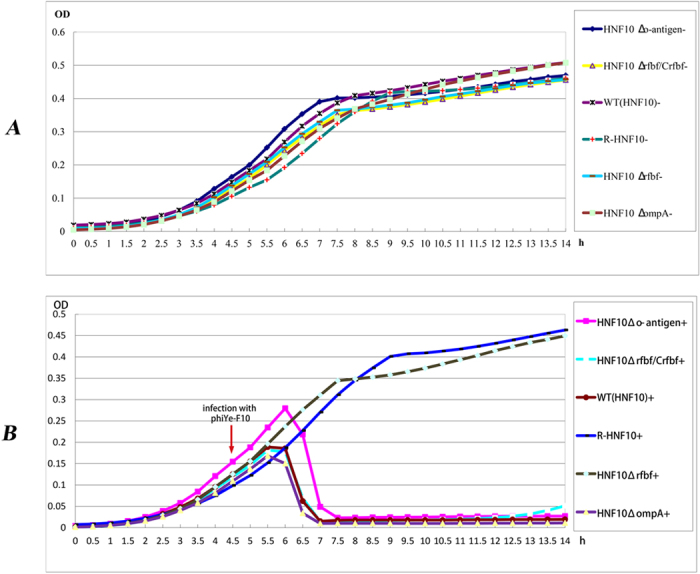
The growth curves of WT, its mutants, and compensation strain. (**A**) The growth curves of strains without phage infection. (**B**) The growth curves of strains with phage infection. Approximately 6 × 10^7^ PFU of phiYe-F10 in 1 ml was mixed with the 10ml bacterial culture (OD600 ≈0.1–0.2), and incubated at 25 °C for 9.5 h. Control cultures were grown without phage infection. The OD_600_ of each group was measured every half hour. At the time indicated by arrow, phage phiYe-F10 was added to the culture.

**Figure 4:**
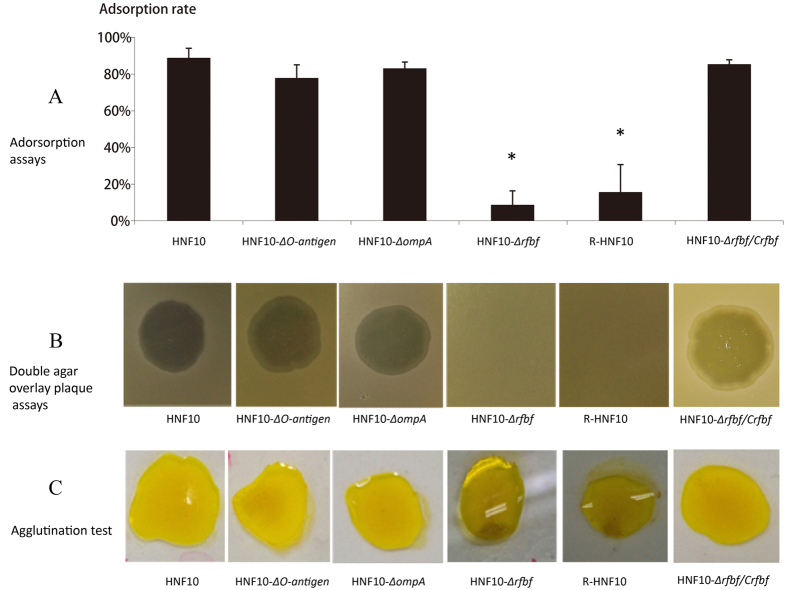
The results of phage adsorption assay, the double-layer plaque assay and Acriflavine agglutination test of WT, its mutants, and compensation strain. (**A**) Adsorption assay of bacteriophage phiYe-F10 to the test strain. Approximately 6 × 10^6^ PFU of phiYe-F10 in 100 μl was mixed with 500 μl samples of bacteria (OD600 ≈1.0). The adsorption rate of each strain was calculated as (Pt′-Pt)/Pt′. Error bars denote statistical variations. Significance was determined by Dunnett T3 test for comparison between the mutant group and the WT group. *P < 0.05. (**B**) The double-layer plaque assay. (**C**) Acriflavine Agglutination test.

The mutants HNF10-Δ*rfbf*, HNF10-Δ*O-antigen* and HNF10-Δ*ompA* were verified by PCR (Supplemental data 2). The WT, knock-out mutant strains, the spontaneous rough mutant R-HNF10 (A spontaneous rough derivative of *Y. enterocolitica* serotype O:3 strain HNF10 carried with virulence-plasmid), and compensation strain HNF10-Δ*rfbf/Crfbf* were all grown to log phase in LB broth at 25 °C with agitation reaching a similar value of OD_600_ for each group. After infecting with phage phiYe-F10 (at 4.5 h in total growth), every half hour the OD was graphed generating a growth curve for each group. The data showed WT, HNF10-Δ*ompA*, HNF10-Δ*O-antigen* and HNF10-Δ*rfbF/Crfbf* were suppressed after infecting with phage phiYe-F10, and then began lysing (Fig. 3B). The OD_600_ value of the WT and HNF10-Δ*ompA* showed a decrease after one hour (at 5.5 h in total growth) with phage phiYe-F10 infection; and then 2.5 hours later (at 7 h in total growth), both WT and HNF10-Δ*ompA* were completely lysed at OD_600_ ≈0. phiYe-F10 adsorbed to the HNF10-Δ*O-antigen* and then began lysing (at 6 h in total growth), with half hour late than WT and HNF10-Δ*ompA* (at 5.5 h in total growth). And 3 h (at 7.5 h in total growth) later, HNF10-Δ*O-antigen* reached complete lysis with an OD_600_ ≈0. The HNF10-Δ*rfbf* and R-HNF10 resisted to phage infection with OD_600_ values of growth curves similar to their control groups (Fig. 3A,B). Consistent with these results, the adsorption assays showed a significant reduction in phage adsorption to R-HNF10 and HNF10-Δ*rfbf* when compared to the adsorption rate of wild-type HNF10 strain, and the difference was statistically significant (Fig. 4A).

### dTDP-rhamnosyl transferase RfbF coding gene *rfbc* deletion in *Y. enterocolitica* resulted in resistance to phage phiYe-F10

The WT, knock-out mutants strains (HNF10-Δ*rfbf*, HNF10-Δ*O-antigen*, HNF10-Δ*ompA*), spontaneous rough mutant R-HNF10, and compensation strain HNF10-Δ*rfbf*/*Crfbf* were tested the sensitivity to the phage with the double-layer plaque assay (Fig. 4B). The results showed the knock-out mutant strain HNF10-Δ*rfbf* and spontaneous rough mutant R-HNF10 were resistant to the phage, producing no plaques and showing a weak positive in the Acriflavine agglutination test; while the WT, HNF10-Δ*O-antigen*, HNF10-Δ*ompA*, and HNF10-Δ*rfbf*/*Crfbf* strains produced plaques and showed negative in the Acriflavine agglutination test (Fig. 4B,C).

## Discussion

In natural environments, bacteria and phage are competing and co-existing with each other. In this competition, the bacteria evolve resistance mechanisms against phage infection. These strategies include blocking receptors or altering receptor structures to prevent phage adsorption, preventing phage DNA entry, digesting phage nucleic acid, inhibiting replication of the phage genome, and cause abortive phage infection. Conversely, bacteriophages are capable of rapid adaptive responses to evolutionary changes in their hosts. As a counter-defensive measure, phages are able to modify their receptor binding proteins, e.g. the tail fiber, to achieve infection and kill the resistant bacterium. For example, *Pseudomonas fluorescens* SBW25 was found to coevolve with its lytic phage phi2 for more than 300 bacterial generations. This co-evolution is probably due to the continuous modification of the bacterial receptors and phage receptor binding protein^36^,^37^.

*Yersinia enterocolitica* is a common foodborne pathogen with O:3 and O:9 as the primary infectious serotypes in most countries^8^,^38^. It was reported that, there were two lytic phages which infected *Yersinia enterocolitica* O:3 had been fully sequenced: phiYeO3-12 (GenBank accession no. AJ251805.1) and vB_YenP_AP5 (GenBank accession no. KM253764.1). The genome sequences of YeO3-12 and vB_YenP_AP5 were submitted to the GenBank databases in 1999 by Pajunen, M.I. and colleagues and in 2014 by Leon-Velarde, C.G. and colleagues, respectively. The two phages were all belonged to the family *Podoviridae*^12^,^14^. In this study, the lytic *Yersinia* phage phiYe-F10, was isolated together with the pathogenic bioserotype 3/O:3 *Y. enterocolitica* HNF-10 from the same swine rectal swab sample. The whole genome sequence of phage phiYe-F10 showed great similarities to *Yersinia* phage PhiYeO3-12 and vB_YenP_AP5, excepted for minor insertions or deletions among the genome sequences in these phages (Fig. 1C). The genomic comparisons indicated the phiYe-F10, PhiYeO3-12 and vB_YenP_AP5 were closely genetically related, and most of their DNA sequences appeared to have descended from a single common ancestral phage. The *Podoviridae* family phage was common in Enterobacteriaceae, which showed the morphologic characteristics of an icosahedral head and a short non-retractable tail when observed under transmission electron microscopy, and the phages were known to have double strands linear genomes with direct terminal repeats^39^,^40^. Currently, the reported *Yersinia enterocolitica* phage were belonged to different families, phage YeO3-12 and vB_YenP_AP5, which specific to infect *Y. enterocolitica* of serotype O:3 belonged to the family *Podoviridae*^12^,^14^; however the temperate phage PY54 (GenBank accession no. AJ564013.1) belonged to the family *Siphoviridae*, which infected *Y. enterocolitica* of serotype O:5 and O:5, 27^11^,^41^; the phage PhiR1-37 (GenBank accession no. AJ972879.2) belonged to the family *Myoviridae*, which infected strain YeO3-R1 (a virulence-plasmid-cured O antigen-negative derivative of *Yersinia enterocolitica* serotype O:3)^42^. The tail fiber protein sequences alignment revealed that the phages belonged to the family *Podoviridae* showed a high similarity and were classified into the same cluster (Fig. 2), suggested that they follow a similar DNA ejection mechanism, with tail fibers adsorbed to the host surface LPS. Although the phage vB_YenP_ISAO8, PhiR1-37 and PY54 displayed lysis for *Y. enterocolitica*, the sequences of tail fiber proteins were clustered into different groups, and the hosts serotypes and phage receptors were different from that of phiYe-F10, phiYeO3-12 and vB_YenP_AP5 (Fig. 2).

Phage phiYe-F10 displayed strict specificity for *Y. enterocolitica* O:3 at 25 °C and 37 °C. Other serotypes (O:8, O:9, O:5, O:5, 27 *et al.*) of *Y. enterocolitica* as well as *Y. pseudotuberculosis* and *Y. pestis* were unaffected by the presence of phage phiYe-F10. Previous research indicated that *Yersinia enterocolitica* phage PhiYeO3-12 and PhiR1-37 used LPS as their receptors^12^,^42^. Here we first confirmed that the dTDP-rhamnosyl transferase RfbF, coded by the *rfbc* gene, played a critical role in synthesizing the phage receptor for phiYe-F10. The 57 phiYe-F10 sensitive *Y. enterocolitica* of O:3 serotype were all carrying the DTDP-rhamnosyl transferase RfbF encoding gene (*rfbc*). The *rfbc* gene, which responsible for the biosynthesis of the O side chain of *Y. enterocolitica*, was chosen for the specific detection target of O:3 serotype. Among the sensitive strains, four strains had different *rfbc* gene with multiple mutation sites compared with the rest of the strains. Although the protein sequence of dTDP-rhamnosyl transferase RfbF changes, the function of the phage receptors was remained and therefore these four strains were still phage sensitive. The *rfbc* gene located within the *rfb* gene cluster encoded the dTDP-rhamnosyl transferase RfbF, which was involved in the dTDP-L-rhamnose biosynthesis. L-Rhamnose is the receptor on the LPS core for the attachment of O polysaccharides^43^, it is an indispensable component of the lipopolysaccharide synthetic pathway^35^,^44^,^45^. *Y. enterocolitica* O:3 O-antigen is a homopolymer of 6-deoxy-L-altrose^26^; the *rfbc* gene regulates the LPS O antigen synthesis pathway. Deletion of dTDP-rhamnosyl transferase RfbF may resulted in the changes or losses of the O antigen, resulting in the failure of phages to bind and infect the strains. Complementation of dTDP-rhamnosyl transferase RfbF deletion mutant recovered its sensitivity to phiYe-F10. Mutation of dTDP-rhamnosyl transferase RfbF retained agglutination with O:3 antiserum, suggested some group modifications of O antigen were responsible for the phage infection but did not affect recognition of the O:3 antiserum.

Deletion of the putative O-antigen biosynthesis protein precursor encoding gene may reduced the infection efficiencies; however, the mutant strain was still phiYe-F10 sensitive. When infected with phage phiYe-F10 at a similar OD in logarithmic phase, the WT strain began to lyse after 1 h and reached complete lysis 2.5 hour later; whereas HNF10-Δ*O-antigen* started lysis at 1.5 h and reached complete lysis 3 hour later. This showed phiYe-F10 adsorbed to HNF10-Δ*O-antigen* slower than WT; but the time needed from the beginning of lysis to complete lysis was the same for the two strains (Fig. 3B). Consequently, we speculated that the putative O-antigen biosynthesis protein precursor played a role in the first steps of virus-host interaction, the deletion of this protein may affected phage adsorption and phage DNA entry, e.g., blocking the phage tail fiber recognition and binding the receptor; or altering the host receptor structure on the bacterial cell surface to prevent phage adsorption, or preventing phage DNA entry. This needs further investigation.

Many studies showed that OmpA was a common phage receptor of Enterobacteriaceae^23^,^31^, but also a common immune protective antigen beyond the V antigen conserved within *Yersinia* species^32^,^33^. Unlike *Y. pseudotuberculosis* and *Y. enterocolitica*, *Y. pestis* had a rough LPS without O antigen where Ail and OmpF were identified to act as the receptors of Yep-phi in addition to the rough lipopolysaccharide of *Y. pestis*^34^. In our study, the OmpA deletion mutant of *Y. enterocolitica* did not affect the binding and lysis of phage phiYe-F10, showing OmpA was not the receptor of phiYe-F10.

A gene altered by artificial or natural mutation will lead to the lack or change of the phage receptor; then alter the phage resistance. In *Y. pestis*, some spontaneous mutations in the core polysaccharide brought about the loss of the LPS core, which resulted in phage resistance^46^. A sequence comparison between the spontaneous resistant strain (R-HNF10) and the WT strain showed the coding genes of dTDP-rhamnosyl transferase RfbF were completely the same. However, R-HNF10 formed obviously rough colonies on agar; and the Acriflavine agglutination test also showed O-antigen deficiency, which further suggested that the phiYe-F10 resistance mechanism resulted from an O-antigen deficiency. Some of the R-HNF10 resistant strains did not agglutinate with O:3 antiserum and monoclonal antibodies, but the others with serotype not changed. This finding suggested small modifications or other changes of O antigen were responsible for the phage infection (binding) but did not affect recognition of the O:3 antiserum. We inferred the epitopes determining serogroup had more and larger distribution than that of phage receptor within the LPS O side-chains.

This study firstly showed the phage receptor of phiYe-F10 was regulated by dTDP-rhamnosyl transferase RfbF which was encoded by the LPS O side-chain synthesis related gene, *rfbc*. Presently, O:3 serotype *Y. enterocolitica* infections occur widely in the world and bring heavy burden to the social community and families^8^,^47^. The phage is proposed as a promising alternative to conventional antibiotics for treating bacterial infections. The emergence and high occurrence of this serotype strain requires in-depth research of the phage specific for *Y. enterocolitica* serotype O:3. The finding of the phage specific for *Yersinia enterocolitica* serotype O:3 and the successful detection of its receptor regulatory protein may provide a foundation for the prevention of infection and transmission of *Y. enterocolitica*.

## Methods

### Bacteriophage isolation and sensitivity test

A lytic phage, named phiYe-F10, specific for *Yersinia enterocolitica* was isolated from a swine rectal swab sample. The phage concentration in the swine rectal swab, was estimated using the plaque assay on *Y. enterocolitica* HNF-10 (Bioserotype 3/O:3, virulence plasmid positive). The phage was isolated together with strain HNF-10 from the same sample in a routine prevalence surveillance for *Yersinia* in China.

From these specimens, phiYe-F10 was chosen for detailed study because of its ability to infect *Y. enterocolitica* strains of serotype O:3. The host range of phiYe-F10 was determined using a double-layer plaque assay at 25 °C and at 37 °C, with one *Y. pestis*, 188 *Y. enterocolitica* and 37 *Y. pseudotuberculosis* belonging to different serotypes. Strains used in the bacteriophage sensitivity test were listed in Table 1. Among the 188 different *Y. enterocolitica*, 183 were widely distributed within 17 provinces of China from 1982 to 2014; and 5 reference strains were provided by H. Fukushima at the Shimane Prefectural Institute of Public Health, Matsue, Japan. The strains were collected from routine monitoring of animals, food and patients with diarrhea (Supplemental data 1).

### Electron microscopy

Filtered phage lysates (about 2 × 10^10^ PFU/mL) were pelleted at 25,000 × g for 1 h at 4 °C, using a Beckman high-speed centrifuge with a JA-18.1 fixed-angle rotor (Beckman, Palo Alto, CA, USA). The phage pellet was washed twice in neutral 0.1 M ammonium acetate. The final phage sediment was re-suspended in 150 μL of SM-buffer supplemented with 5 mM CaCl_2_. Samples were then deposited onto a carbon-coated Formvar film on copper grids, and stained with 20 μl of 2% potassium phosphotungstate (PT, pH 7.2). Get rid of the dye with filter paper, air dried, and examined under a TECNAI 12 transmission electron microscope (FEI, Hillsboro, OR, USA) at 120 KEv. Images were collected and analyzed using Digital Micrograph™ Software (Gatan, Pleasanton, CA, USA).

### The Genome sequencing of phage DNA, assembly and bioinformatics analysis

A random “shotgun” library was constructed using a fast nebulization method^48^ and the fragments were ligated to the vector pUC118 (TaKaRa Code: 3322, pUC118 HincII/BAP). Cycle sequencing reactions from the plasmid inserts were performed using the ABI PRIS Mw Big Dye Terminator Cycle Sequencing Kit (Applied Biosystems) with a Gene Ampw PCR System 9700 (Applied Biosystems). Sequence reactions were analyzed using an ABI PRISMTM 3730XL DNA Analyzer (Applied Biosystems). Sequencing continued until an eight-fold coverage of the sequenced plasmid was attained. Assembly of the sequences was performed using the SeqMan module of the Lasergene software (DNASTAR Inc.). Persisting gaps were closed using primer-walking sequencing of the genomic DNA.

A dot plot comparison and graphical comparison were conducted using BLAST 2.25 and displayed using ACT^49^. The minimum score cutoff was 100 and the minimum identity cutoff was 50%.

Sequences of tail fiber proteins were collected from NCBI (6 phages for *Y. enterocolitica*, 1 phage for *Y. pestis*, and the *Escherichia coli* phages T7 and T3), the phylogenetic tree was generated using the neighbor-joining method implemented in MEGA6. Bootstrap values representing the percent in 1,000 replicates were shown in the tree.

### Bacteria, plasmids, and growth media

The *Y. enterocolitica* wild type strain HNF10 was isolated from a swine rectal swab sample from Henan province. R-HNF10 was a spontaneous rough mutant of HNF10. The knock-out mutants HNF10-Δ*O-antigen*, HNF10-Δ*ompA*, and HNF10-Δ*rfbf* were constructed in this work. The strains and plasmids used for gene cloning and mutation were listed in Table 3. The serotypes, biotypes, and pathogenicity of these strains were determined as previously describe^8^,^50^. The *E. coli* and *Y. enterocolitica* strains were incubated at 37 °C and 28 °C, respectively. Solid and soft agar media contained 2.0% and 0.5% (w/v) agar, respectively. Antibiotics were used at the following concentrations: kanamycin: 50 μg/ml for agar plates and 100 μg/ml for broth; chloramphenicol, 34 μg/ml; cefsulodin, 15 μg/ml; and novobiocin, 2.5 μg/ml.

### Construction of HNF10-Δ*rfbF*, HNF10-Δ*O-antigen* and HNF10-Δ*ompA* knock-out mutants

The deletion mutants were constructed using homologous recombination with the suicide plasmid pDS132 51. To obtain the knock-out mutants, the corresponding two sets of primers were used to amplify two different fragments respectively. The p1 and p2 primers amplified the upstream region of genes, the P3 and P4 primers amplified the downstream region of genes (Table 4). pDS132-*O-antigen*, pDS132-*ompA* and pDS132-*rfbf* were generated using the In-Fusion HD Cloning Kit (Clontech) following the manufacturer’s instructions. The recombinant plasmids were induced into the competent cell S17λpir^52^, and then mobilized into *Y. enterocolitica* HNF10 through biparental conjugation. Transconjugants were selected after growth on LB plates containing *Yersinia* complement (cefsulodin and novobiocin) and chloramphenicol. Bacteria from individual colonies were pooled and allowed to grow in LB without antibiotic overnight at 25 °C. Bacterial cultures were serially diluted in LB without NaCl containing 10% sucrose; the plates were incubated at 25 °C. The recombinants that survived in the 10% sucrose were examined for their antibiotic resistance. The appropriate replacement of the wild-type alleles by the mutants was confirmed using PCR and sequencing.

### Complementation of mutations

Primers *rfbf*CF and *rfbf*CR, incorporating *NdeI* and *SacI* restriction sites (Table 4), were used to amplify the ORF of *rfbc*, including the 904-bp region in the HNF10 genome. The amplicon was digested with *NdeI* and *SacI* (New England BioLabs) and ligated into the *NdeI*- and *SacI*-digested plasmid pSRKKm (D3050; TaKaRa, Japan)^53^, producing plasmid pSRKKm–*rfbf* (the *rfbc* ORF cloned into pSRKKm, KmR); introduced into the competent cell S17λpir; and then mobilized into *Y. enterocolitica* HNF10-Δ*rfbf* using biparental conjugation. The recombinant clones HNF10-Δ*rfbf*/*Crfbf* were confirmed using PCR and sequenced to ensure they contained the correct insert sequence.

### Phage adsorption assays

The WT, R-HNF10, HNF10-Δ*rfbf*, HNF10-Δ*O-antigen* HNF10-Δ*ompA* knock-out mutant strains and compensation strain HNF10-Δ*rfbf*/*Crfbf* were cultured overnight on BHI at 25 °C. Approximately 6 × 10^6^ PFU of phiYe-F10 in 100 μl was mixed with 500 μl samples of bacteria (OD_600_ ≈1.0). The suspension was incubated at room temperature for 5 min and centrifuged at 16,000 × g for 3 min, after which the phage titer remaining in the supernatant was determined as Pt. BHI was used as a non-adsorbing control in each assay, and the phage titer in the control supernatant was set to Pt′. The adsorption rate of each strain was calculated as (Pt′-Pt)/Pt′× 100%, each stain was repeated three times.

### Agglutination test

The WT, R-HNF10, knock-out mutant strains and compensation strain were agglutinated using concentrations of Acriflavine. This procedure offered a simple way to distinguish LPS defective bacteria. Bacteria were suspended in 0.2% Acriflavine solution, the LPS defective strains agglutinated, whereas the LPS positive strains showed no agglutination^54^,^55^.

### The growth characteristic of WT, mutant strains and compensation strain

The WT, R-HNF10, HNF10-Δ*rfbf*, HNF10-Δ*O-antigen* HNF10-Δ*ompA* and HNF10-Δ*rfbf*/*Crfbf* were grown overnight and 1:100 diluted in fresh LB medium at the zero time point. After 4.5 h culture, 1 ml of phiYe-F10 (6 × 10^7^ PFU/ml) was mixed with the bacterial culture (10 ml), and incubated at 25 °C for 9.5 h. Control cultures were grown without phage infection. The OD_600_ of each group was measured every half hour. Each group was repeated three times.

### Ethics statement

The sample collection and detection protocols were carried out in accordance with relevant guidelines and regulations. All experimental procedures were approved by the Ethics Review Committee [Institutional Review Board (IRB)] of National Institute for Communicable Disease Control and Prevention, Chinese Center for Disease Control and Prevention. Signed informed consent was obtained from all study participants.

## Additional Information

**Accession codes:** The annotated genome sequence for the phage phiYe-F10 was deposited in the NCBI nucleotide database under the accession number KT008108.

**How to cite this article**: Liang, J. *et al.* DTDP-rhamnosyl transferase RfbF, is a newfound receptor-related regulatory protein for phage phiYe-F10 specific for *Yersinia enterocolitica* serotype O:3. *Sci. Rep.* **6**, 22905; doi: 10.1038/srep22905 (2016).

## Supplementary Material

Supplementary Dataset 1

Supplementary Information

## Figures and Tables

**Table 1:** Bacteriophage sensitivity test*.

Y. enterocolitica			Y. pseudotuberculosis		
Serotype	Resistant No.	Sensitive No.	Serotype	Resistant No.	Sensitive No.
O:10K	1	0	O:10	1	0
O:19, 8	1	0	O:5a	1	0
O:20	1	0	O:9	1	0
O:44	1	0	O:2a	2	0
O:4, 32	2	0	O:6	2	0
O:5, 27	3	0	O:8	2	0
O:5	10	0	O:15	3	0
O:8	13	0	O:1a	3	0
O:9	34	0	O:14	4	0
O:3	0	57	O:1b	4	0
SA	5	0	O:3	5	0
UD	60	0	O:4b	5	0
			NT	4	0
Total	131	57	Total	37	0

*Note: The one *Yersinia pestis* used in this study is not listed in this table.

UD: Undetermined; NT: Not typable; SA: self-agglutingating.

**Table 2:** The characteristics of the 57 phage sensitive *Y. enterocolitica* strains of O:3 serotype.

Serotype	Genotype	Biotype				Sensitive rate
		1A	3	4	5	(%)
O:3	*rfbc*+*, ail*+*, ystA*+*, ystB*−*, yadA*+*, virF*+	0	36	3	4	75.44%
	*rfbc*+*, ail*+*, ystA*+*, ystB*−*, yadA*−*, virF*−	2	3	3	0	14.04%
	*rfbc*+*, ail*−*, ystA*−*, ystB*+*, yadA*−*, virF*−	2	0	0	0	3.51%
	*rfbc*+*, ail*−*, ystA*−*, ystB*−*, yadA*−*, virF*−	4	0	0	0	7.02%

**Table 3:** Strains and plasmids used for gene cloning and mutation in this study.

Strains and plasmids	Description or comments	Source orreference
Strains		
* Y. entercolitica* HNF10	BioSerotype 3/O:3 with pYV plasmid, isolated in Henan, China from a swine rectal swab	This study
* *HNF10-Δ*O-antigen*	Strain HNF10 with a deletion of gene for putative O-antigen biosynthesis protein precursor	This study
* *HNF10-Δ*ompA*	Strain HNF10 with a deletion of *ompA* gene	This study
* *HNF10-Δ*rfbf*	Strain HNF10 with a deletion of *rfbc* gene	This study
* *HNF10-Δ*rfbf/Crfbf*	*rfbc* complementation of HNF10-Δ*rfbf*	
* *R-HNF10	Spontaneous rough mutant of HNF10	This study
* *S17 λpir	λ-pir R6K (thi thr leu ton lacY supE recA::RP4-2Tc::Mu)	52
Plasmids		
* *pDS132	Conditionally replicating vector; R6K origin, mobRK4 transfer origin, sucrose-inducible sacB, CmR	51
* *pDS132-*O-antigen*	containing a fusion fragment of upstream and downstream of the *O-antigen* gene	This study
* *pDS132-*ompA*	containing a fusion fragment of upstream and downstream of the *ompA* gene	This study
* *pDS132-*rfbf*	containing a fusion fragment of upstream and downstream of the *rfbc* gene	This study
* *pSRKKm	Expression vector containing lac promoter and lacIq, KmR	53
* *pSRKKm -*rfbf*	*rfbc* orf cloned into pSRKKm, KmR	This study

**Table 4:** Primers used in this study.

Primer	Sequence (5′–3′)
Construction of mutants	
* rfbf* p1	AAAGGATCGATCCTCTAGAGGCGATCCTTATGGGTAC
* rfbf*p2	GCTAGAAACTCACTAGAAAGATTTAAGAGACCGAAAA
* rfbf*p3	TTTTCGGTCTCTTAAATCTTTCTAGTGAGTTTCTAGC
* rfbf*p4	CCCGGGAGAGCTCGATATCGCAACATCACGGAATCTT
* ompA* p1	AAAGGATCGATCCTCTAGAGACAGAAATTGAGTCGTTTAT
* ompA* p2	GTTTTTTACGGAAGCAGTAAACTTTACGCCTCGTTATC
* ompA* p3	GATAACGAGGCGTAAAGTTTACTGCTTCCGTAAAAAAC
* ompA* p4	CCCGGGAGAGCTCGATATCTAGTAGGGTGAAATTCT
* Oag* P1	AAAGGATCGATCCTCTAGAGGAGGTTGTTACTGTTAC
* Oag* P2	AAGAATTTTTCTCTTCATATAATTTCCCTCCAAGAGA
* Oag* P3	TCTCTTGGAGGGAAATTATATGAAGAGAAAAATTCTT
* Oag* P4	CCCGGGAGAGCTCGATATCGTTGGTGCGTCACTAAGA
Complementation of mutation HNF10-Δ*rfbf*	
* rfbf*CF	AATC*CATATG*AATGCATCGCAATATTC
* rfbf*CR	AATC*GAGCTC*TTAATTTAACTTACCTG

Note: Restriction endonuclease sites are underlined.
