# Supplementary figures and images for: DTDP-rhamnosyl transferase RfbF, is a newfound receptor-related regulatory protein for phage phiYe-F10 specific for Yersinia enterocolitica serotype O:3
Source: Sci Rep. 2016 Mar 11;6:22905. doi: 10.1038/srep22905 (PMC4786787; doi:10.1038/srep22905)

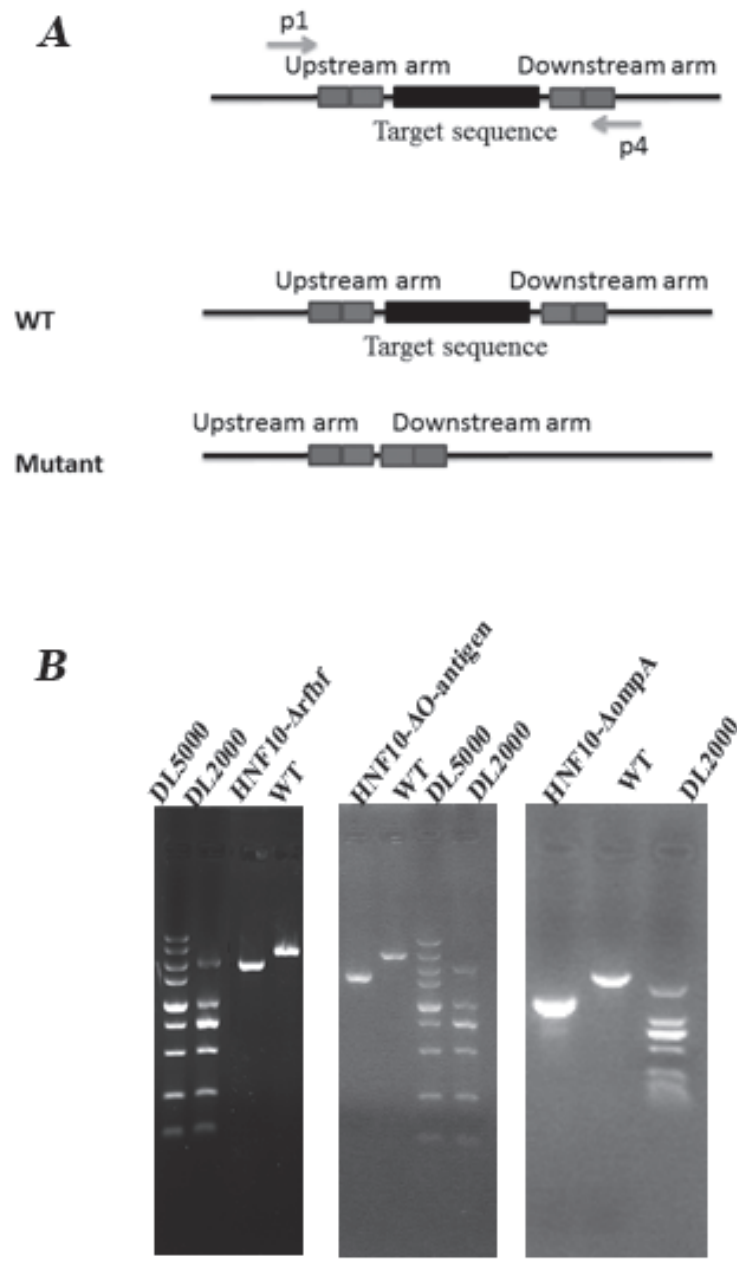

Supplemental data 2: PCR verification of the deletion mutant strains.

Supplement: Supplementary Information [file srep22905-s2.pdf]
